# Increased Drinking following Social Isolation Rearing: Implications for Polydipsia Associated with Schizophrenia

**DOI:** 10.1371/journal.pone.0056105

**Published:** 2013-02-18

**Authors:** Emily R. Hawken, Nicholas J. Delva, Richard J. Beninger

**Affiliations:** 1 Centre for Neuroscience Studies, Queen’s University, Kingston, Canada; 2 Department of Psychiatry, Queen’s University, Kingston, Canada; 3 Department of Psychiatry, Dalhousie University, Halifax, Canada; 4 Department of Psychology, Queen’s University, Kingston, Canada; Prince Henry’s Institute, Australia

## Abstract

Primary polydipsia, excessive drinking without known medical cause, is especially associated with a diagnosis of schizophrenia. We used animal models of schizophrenia-like symptoms to examine the effects on schedule-induced polydipsia: post-weaning social isolation rearing, subchronic MK-801 treatment (an NMDA-receptor antagonist) or the two combined. Male, Sprague-Dawley rats reared in groups or in isolation beginning at postnatal day 21 were further divided to receive subchronic MK-801 (0.5 mg/kg twice daily) or saline for 7 days beginning on postnatal day 62. Following a 4-day withdrawal period, all groups were trained on a schedule-induced polydipsia paradigm. Under food-restriction, animals reared in isolation and receiving food pellets at 1-min intervals developed significantly more drinking behavior than those reared with others. The addition of subchronic MK-801 treatment did not significantly augment the amount of water consumed. These findings suggest a predisposition to polydipsia is a schizophrenia-like behavioral effect of post-weaning social isolation.

## Introduction

Primary polydipsia, the clinical term for ‘excessive water drinking’, is commonly associated with chronic psychiatric illness, reportedly occurring in a large subset of hospitalized patients with schizophrenia [1]. Characterized by a delayed onset, polydipsia often emerges several years following the first psychotic episode and is correlated with measures of increased cognitive dysfunction [2] and greater severity of psychotic illness [3]. As patients with polydipsia associated with schizophrenia have significantly increased rates of mortality [4], identifying and managing psychogenic polydipsia in the clinical setting is important. However, outside of restricting the patient’s daily fluid intake, few pharmacological treatments have shown reliable control of excessive water drinking in this population.

The development of novel therapeutics is often aided by understanding the neuropathology of a behavior or disease, yet little is known about the neuronal mechanisms associated with polydipsia in schizophrenia. Converging evidence implicates the hippocampus. Polydipsic-normonatremic and polydipsic-hyponatremic patients with schizophrenia show altered neuroendocrine regulation (vasopressin, adrenocorticotropin and oxytocin; [5], [6], [7]). Because the hippocampus plays a modulatory role in vasopressin regulation, some hypothalamic-pituitary-adrenal responses to stress, and glucocorticoid feedback [7], [8], [9], these findings may suggest a hippocampal deficit in this population. Reports of reduced anterior hippocampal volume [10] in patients with hyponatremia associated with polydipsia support this idea. Furthermore, dysfunction of hippocampal neurons is a signature pathological feature of schizophrenia [11], [12] suggesting a role for the pathology of schizophrenia in the development of polydipsia. Whether or not hippocampal neuropathology is causative of polydipsia associated with schizophrenia has yet to be determined.

Current animal models that mimic some of the symptoms associated with schizophrenia (e.g., affect blunting, social withdrawal, impairment of memory and executive function) have potential to reveal an etiological link between polydipsia and schizophrenia (for review see [13]). Rats treated twice daily for 7 days (i.e., subchronically) with glutamate N-methyl-D-aspartate (NMDA) receptor antagonists (e.g., MK-801) show a number of schizophrenia-like symptoms including increased locomotor responses to amphetamine [14], decreased prefrontal cortical dopamine release [15] and decreased GABA interneurons in the hippocampus [16]. Paired with a paradigm that induces excessive water consumption (food-restricted rats intermittently fed small amounts, dubbed schedule-induced polydipsia or SIP [17]), subchronic MK-801 treated animals showed increased excessive drinking behavior [18].

Unlike subchronic MK-801, post-weaning social isolation rearing (IR) is a non-pharmacological animal model of schizophrenia-like symptoms. Socially isolating rats from weaning (postnatal day [P] 21) through to sexual maturation leads to impaired sensorimotor gating, social withdrawal and impaired cognitive flexibility [19]. The effects of social isolation show a critical period, where isolation between P25 and P45 days followed by group housing reveals irreversible effects on some behaviors [20], [21], [22]. Furthermore, social isolation during development has an effect on neurochemistry [23], mimicking alterations seen in schizophrenia such as evidence for decreased GABA neurotransmission in the hippocampus and prefrontal cortex [24], suggesting that IR may like-wise augment the development of polydipsic behavior.

Here we hypothesized that post-weaning IR will increase drinking in a SIP paradigm. Additionally we investigated the effects of a combined ‘double-hit’ animal model (IR plus subchronic MK-801 treatment) hypothesizing that it will yield a more robust effect on subsequent development of SIP behavior than either insult alone.

## Experimental Methods

### Subject Housing

Male Sprague-Dawley rats were obtained at weaning (P21; Charles River, QC). Upon arrival, rats were randomly assigned to housing either in groups of four (group reared, GR) or alone (IR; Table 1), in clear Plexiglas cages (45×23×20 cm deep for IR rats and 47×37×20 cm deep for GR rats). The floors were lined with bedding (Beta Chip, NEPCO, Warrensburg, NY) and the cages were located in a climate-controlled colony room (21±1°C; humidity 40–70%) on a reversed 12-hr light/dark schedule (lights off at 0700 hr). Animals had free access to food (LabDiet rodent feed #5001, PMI Nutrition International, Brentwood, MO) and water. Rats were treated in accordance with the Canadian Council on Animal Care regulations and the behavioral protocols and this study received approval from the Queen’s University Animal Care Committee.

**Table 1:** Experimental design: number of rats assigned to each group for each experiment.

Housing+Treatment	Experimental Paradigm *SIP*	Control Paradigm *Free Feed*
Experiment 1:		
IR+no injection	7	n/a
GR+no injection	8	n/a
Experiment 2:		
IR+MK-801	8	8
IR+Saline	8	8
GR+MK-801	12	8
GR+Saline	12	8

Abbreviations: GR, group reared; IR, isolation reared; n/a, no animals in these groups; SIP, schedule-induced polydipsia.

### Drug Treatment

At 62 days, rats were further assigned to MK-801 injected (Sigma, Oakville ON; dissolved in saline, 0.5 mg/kg, i.p.), saline injected (1.0 ml/kg) or no injection groups (Table 1). Injected rats received twice-daily injections (at 0900 hr and 2100 hr) for seven days. A four-day washout followed and food restriction (1 hr *ad lib* per day) began and continued for the study duration.

### Apparatus

Four commercially built (Med Associates Inc., St. Albans, VT) operant chambers (30.5×24.1×21 cm) were used. Specifications are identical to and summarized in Hawken et al. [18].

### Behavioral Testing

Animals completed two hours of daily testing for 21 days, in either schedule-induced polydipsia (SIP; experimental) or free feed (control) paradigms (Table 1). Weights were recorded before testing.

In the experimental paradigm, a pellet was automatically dispensed every min (120 pellets/120 min) during 2-hr sessions. Each pellet consisted of a 45 mg dustless precision food pellet (Bio-serv, Frenchtown NJ). In the control paradigm, 120 pellets were instead freely available in a dish. Over each session, groups had free access to the drinking spout. Animals were tested in groups of 4 (one per chamber) counterbalanced across testing boxes and time of testing in daily morning and afternoon sessions. The volume of water consumed per session was calculated by measuring before and after weights of water bottles. Following testing, animals were returned to their home cages and fed for 1 hr.

### Experimental Design

Table 1 summarizes the experimental design. In Experiment 1, IR and GR animals did not receive injections before completing the experimental paradigm. In Experiment 2, IR and GR animals received either MK-801 subchronic treatment or saline and completed either experimental or control paradigms. An extra 4 rats were included in the GR-MK and GR-SAL to reduce possible Type II error.

### Statistical Analysis

Analyses were performed using SPSS version 17.0 (Chicago, IL). Animals were deemed to have achieved SIP when they consumed at least 15 ml of water per 2-hr session for 3 consecutive days [18].

Experiment 1: Two-way repeated measures analysis of variance (ANOVA; housing × day) evaluated main effects and interactions.

Experiment 2: Four-way repeated measures ANOVA (housing×treatment×day×paradigm) examined main effects and interactions. Three-way repeated measures ANOVAs examined significant interactions (housing×treatment×day). A 2-way repeated measures ANOVA examined possible effects of handling for injections by comparing rats in the experimental treatment groups that were saline treated (GR and IR) to groups in experiment 1 that were GR and IR but not injected. Chi-square tests examined proportions of rats achieving the SIP criterion in experimental groups.

ANOVA was used to compare mean body weights of groups before behavioral testing and to further explore significant interactions in the above experiments. When repeated measures were analyzed, the Huynh-Feldt statistic was used to evaluate within-subject effects, although unadjusted degrees of freedom are reported.

## Results

### Experiment 1

**Figure 1:**
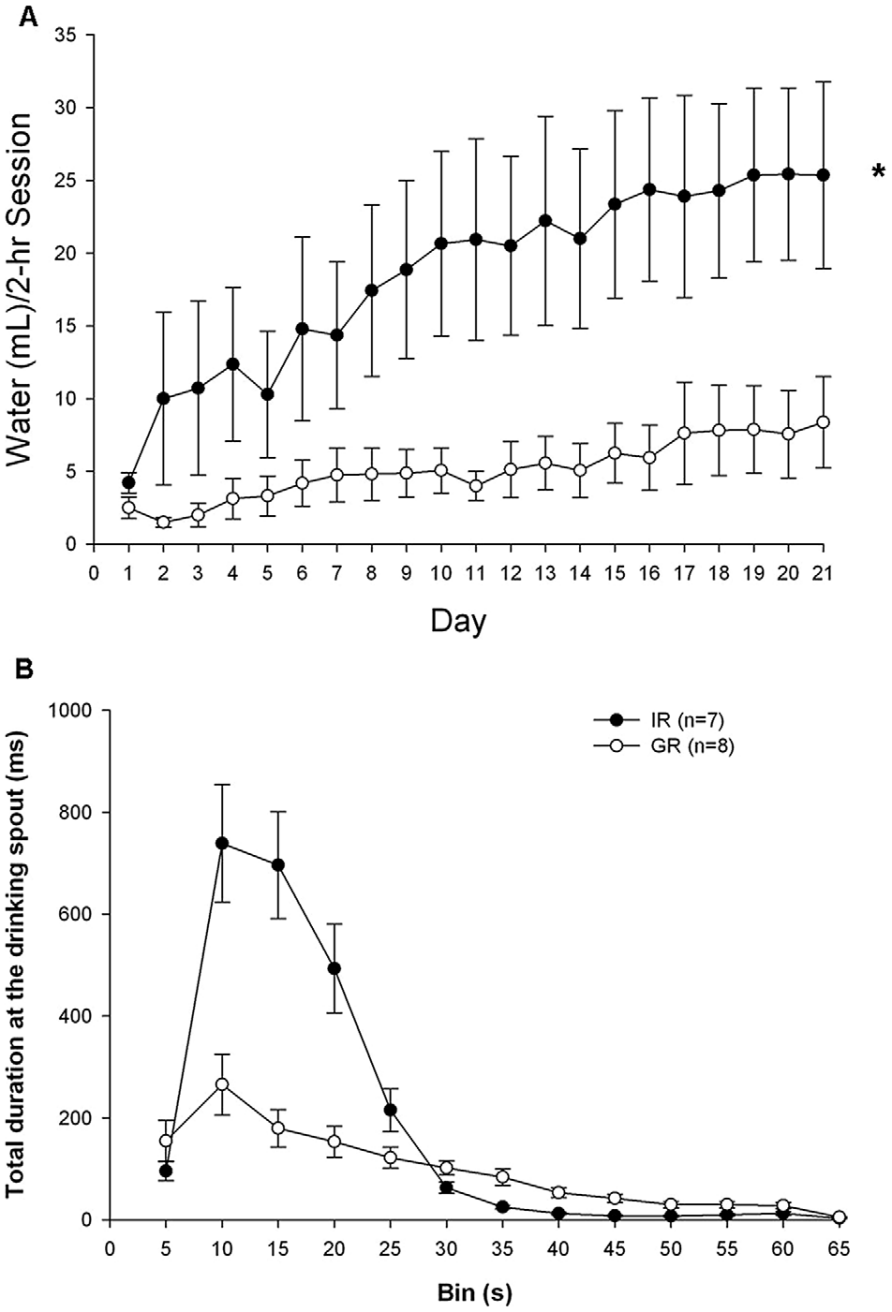
The effects of social isolation on schedule induced polydipsia (SIP). (A) Isolation rearing (IR) housing significantly increased drinking in the schedule induced polydipsia paradigm across days. Mean (±SEM) daily water drinking during the 2-hr testing session for IR and group reared (GR) groups in the experimental paradigm are shown. IR animals were not different from GR animals on Day 1 of testing (F[1], [14] = 2.8, p = 0.12) but on Day 21 drank more water than the GR group (F[1], [14] = 6.2, p = 0.04). (B) Intermittent food presentation increased time spent at the drinking spout. Mean (±SEM) duration (ms) spent at the drinking spout during the inter-pellet interval (1 min; measured in 5-s bins) for IR and GR groups. Both groups increased spout time to a maximum within 10-s following pellet delivery and decreased time toward the end of the interval. This pattern was more pronounced in the IR group suggesting increased drinking behavior. *Two-way analysis of variance, significant interaction of day×housing (F[20,260] = 2.76, p = 0.047).

Animals reared in isolation drank substantially more during daily 2-hr sessions across days than animals raised within a group (Fig. 1A); two-way ANOVA revealed a significant main effect of day (F[20,260] = 8.74, *p*<.001) and housing (F[1], [13] = 6.32, *p = *.026) and a significant interaction of day×housing (F[20,260] = 2.76, p = 0.047). Furthermore, more IR animals (4/7; 57%) developed SIP compared to GR (1/8; 12.5%). Body weight was not different between groups at the start of SIP training (IR = 320 g ±15; GR = 347 g ±16; F[1], [13] = 1.40, p = 0.26).

Figure 1B illustrates the postprandial drinking pattern in the IR animals that is characteristic of SIP. Quantified as the total duration of time spent at the drinking spout, the significantly greater drinking of the IR animals (Fig. 1A) took place in the 15–20 s following consumption of the pellet further illustrating that more SIP was achieved in the IR group.

### Experiment 2

**Figure 2:**
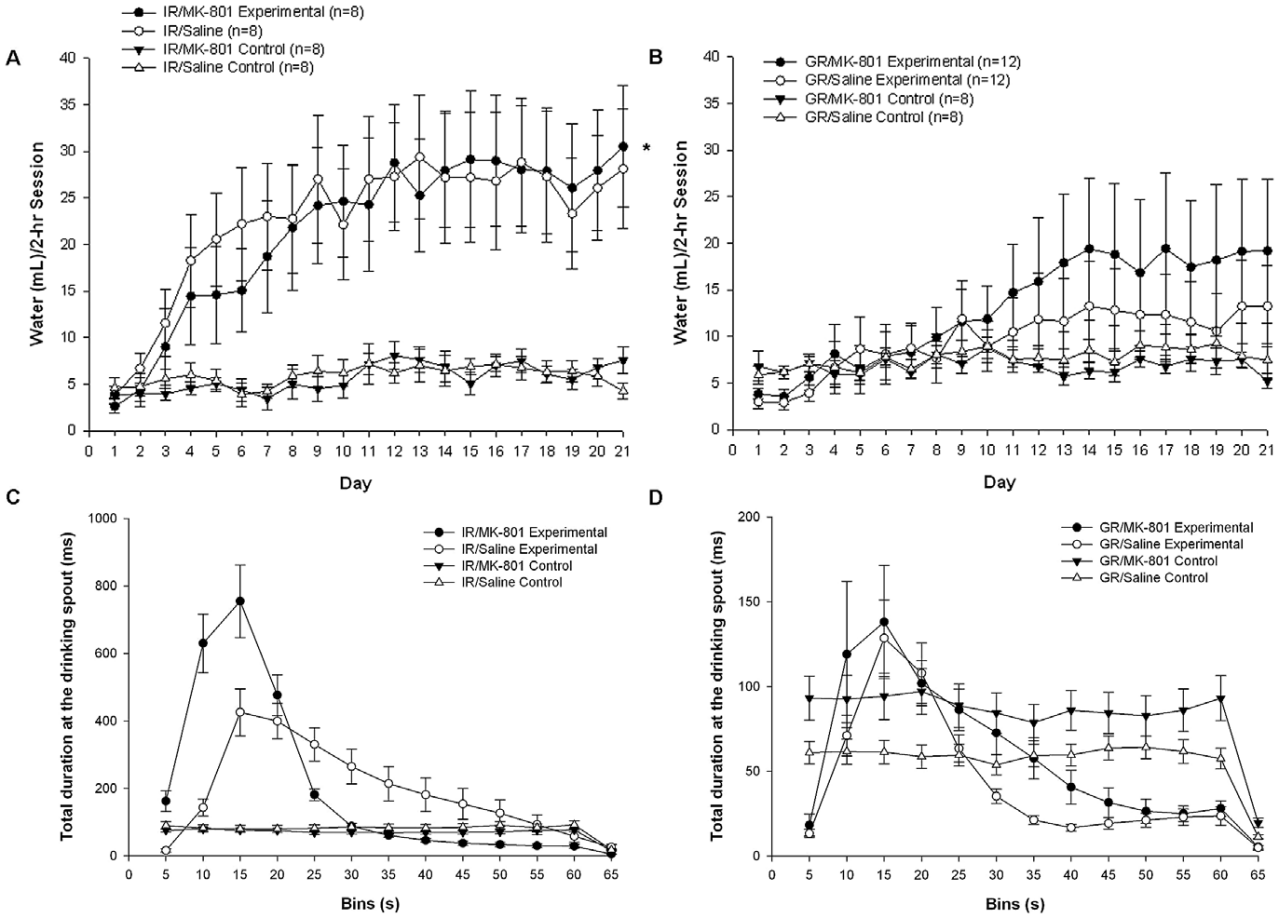
The effects of social isolation and MK-801 treatment on schedule induced polydipsia (SIP). (A) Isolation rearing (IR) significantly increased drinking in schedule induced polydipsia paradigm across days. Daily mean water drinking during the 2-hr testing session for MK-801 and Saline treated IR (A) and group reared (B; GR) animals in the experimental and control paradigms are shown. (C) IR and (D) GR treated groups receiving one pellet a minute (experimental paradigm) showed the postprandial drinking characteristic of SIP, as evidenced by the total duration of time spent at the drinking spout. Note the difference in the scale on the y-axis in (C) and (D) as the GR animals drank less. Spout time for experimental groups increased immediately after pellet delivery, increased to a maximum at 15-s and then declined toward the end of the 60-s interval. Animals in the control paradigm did not show this pattern. IR rats showed a longer duration at the spout throughout the inter-pellet interval consistent with increased drinking behavior. *Analysis of variance revealed a significant main effect for housing (F[1], [36] = 5.50, p = 0.025).

Animals exposed to intermittent pellet presentation (experimental paradigm) drank increasingly more over days than those in the control paradigm (Fig. 2A, 2B); a four-way ANOVA showed a significant main effect of day (F[20,1260] = 15.2, p<0.001) and paradigm (F[1,63] = 15.3, p<0.001), significant interactions of day × paradigm (F[20,1260] = 10.0, p<0.001) and housing×paradigm (F[1,63] = 5.46, p = 0.023).

Three-way ANOVA of the SIP paradigm indicated that the addition of subchronic MK-801 treatment did not induce a “doubling” of the acquisition of SIP behavior but that SI alone contributed to the observed increase in SIP (Fig. 2A) with significant main effects of day (F[20,720] = 16.9, *p*<0.001) and housing (F[1], [36] = 5.50, *p = *0.025). Table 2 reports the proportion of animals that achieved SIP in each group (drinking more than 15 ml per 2-hr session on 3 consecutive days): collapsed across treatment, a chi-square test of independence showed significantly more IR then GR animals developed SIP (*X^2^*[1, N = 40] = 9.70, p = 0.002). IR failed to drink more than GR animals in the control paradigm: 3-way ANOVA examining within-paradigm effects of treatment and housing on daily session drinking revealed only a main effect of day (F[20,540] = 6.6, p = 0.001). While control animals not subjected to the 1-min intermittent food schedule increased drinking over days, all failed to develop SIP.

**Table 2:** Proportion of animals that achieved schedule-induced polydipsia (drinking at least 15 ml per 2-hr session on 3 consecutive days) collapsed across MK-801 treatment in experiment 2.

	MK-801	SALINE
Isolation reared	6/8 (75%)	6/8 (75%) *
Group reared	4/12 (33%)	2/12 (17%)

* Significantly more isolation-reared animals (collapsed across treatment) achieved schedule-induced polydipsia than group-reared animals, Chi-square test, p<0.01.

Figures 2B and 2C further demonstrates how developing SIP within each session was dependent on the SIP paradigm as well as the housing condition: mean duration of time at the drinking spout during 5-s bins of the 60-s inter-pellet interval for the IR groups (Fig. 2C) and the GR groups (Fig. 2D) averaged over days revealed the same pattern of postprandial drinking as that seen in experiment 1 (Fig. 1B).

There were no differences in mean (±SEM) body weights among rearing groups (IR-MK-801 = 380±14 g; GR-MK-801 = 387±4.5 g; IR-Sal = 383±5.8 g; GR-Sal = 395±7.5 g) at the start of SIP training (housing: F[1], [36] = 1.40, p = 0.24), nor were there any effects of injection handling (Fig. 1 and Fig. 2A; 2-way ANOVA, housing [GR×IR] ×injection [Saline×no injection]; F[1], [31] = 1.07, p = 0.31) that would confound the effect of IR on SIP behavior.

## Discussion

This study examined polydipsia in the context of animal models of schizophrenia-like symptoms. Animals exposed to an excessive drinking (SIP) paradigm following post-weaning social isolation, subchronic MK-801 or the two together (‘double-hit’) revealed that rearing animals in isolation significantly augmented drinking behavior. Isolating adult animals that have been group reared does not produce significant amounts of SIP behavior [18]. Unlike our previous findings [18], blocking NMDA receptors with subchronic MK-801 treatment alone did not significantly increase drinking behavior nor add or synergize with the effects of isolation rearing on SIP. These findings suggest that post-weaning social isolation, a putative animal model of schizophrenia-like symptoms, may lead to polydipsia.

Exaggerated drinking patterns were apparent in experimental groups compared to control groups that were not exposed to the intermittent food delivery schedule, as observed by others previously [17], [25]. SIP, in which animals have been observed to drink more in a single 2-hr session than the amount they consume daily [25], is considered an adjunctive behavior [26] that is non-regulatory in nature [27]. In humans, primary polydipsia has been observed to be ‘excessive,’ ‘persistent’, ‘non-regulatory’, and ‘without physiologic cause’ [1] and in this way is similar to SIP. This face-validity makes SIP potentially useful as a model of various neuropsychiatric disorders related to the impulsive-compulsive spectrum disorders [28]. Accordingly, SIP has been proposed as a model for obsessive compulsive disorder (OCD; [29], [30], [31]). Repetitive, obsessive-compulsive behaviors show significant co-morbidity in schizophrenia patients [32], [33], [34], [35] and there is growing evidence to suggest a complex interaction between OCD and schizophrenia-spectrum disorders [36], [37]. However, while OCD treatments (e.g., selective serotonin reputake inhibitors) have been shown to successful diminish SIP (for review see Monero and Flores [31]), they have not been reported to improve polydipsia in schizophrenia. Regardless, the SIP paradigm studied in the context of a schizophrenia framework shows promise as a valid animal model of polydipsia observed in schizophrenia.

The mechanisms by which social isolation exacerbates SIP behavior is unclear. SIP is consistently reduced by acute treatments with dopamine (DA) receptor agonists or antagonists [38], [39], [40], [41]. Furthermore, D2-like receptor binding was increased in the nucleus accumbens (NAc), medial prefrontal cortex, amygdala and ventral tegmental areas in animals that were high drinkers in the SIP paradigm and D1-like receptors were decreased in the same areas [42]. This suggests that an imbalance of DA receptor activation may facilitate abnormal drinking. Post-weaning social isolation also increased DA activity in the NAc in adults [43]; for review see [23]. Evidence of increased DA activity following amphetamine treatment has also been reported in other neurodevelopmental animal models of schizophrenia-like symptoms that specifically disrupt hippocampal GABAergic function (e.g., methylazoxymethanol acetate [MAM]-treated rats) resulting in hyperactivity of some cortical circuits [44].

Individually, social isolation and subchronic NMDA-receptor antagonism may alter GABAergic neurons to produce schizophrenia phenotypes. Rodents isolated throughout development show reduced dendritic arborization (and thus fewer synapses; [45]) and deficits in adulthood of both parvalbumin- (PV+) and calbindin–positive immunoreactive GABA interneurons in the hippocampus [35], [46] thought to regulate glutamatergic pyramidal cell activity. Normal GABAergic function is critical for activity-dependent modeling of the glutamate system during neural development [47], [48]. Suppression of GABA-mediated inhibitory synaptic transmission [24] in IR animals has been suggested by Hickey et al. [24] who found increased GABA transporter 1 (GAT-1) activity in the frontal cortex and hippocampus and GABA(A) receptor expression only in the former. Like social isolation, pharmacologically reducing NMDA function before sexual maturation prevents normal development of GABA inhibitory circuits resulting in reduced parvalbumin levels and excessive cortical excitability [48]. Hickey et al. [24] demonstrated that MK-801 only increased GABA(A) receptor expression in the hippocampus. A loss of GABA interneurons specific to the hippocampus has also been reported following MK-801 treatment [16]. While PV+ interneuron dysregulation has been implicated in the development of schizophrenia, the exact cause and time course of interneuron dysfunction and loss due to post-weaning isolation and MK-801 treatment is unknown.

The above findings might suggest that a ‘double-hit’ model could have more robust effects on behavior than either insult alone. Supporting this hypothesis, Lapiz et al. [49] Simpson et al. [50] and Hickey et al. [24] found enhanced locomotor hyperactivity to a novel environment in IR animals following subchronic phencyclidine or and MK-801 treatment. However, our ‘double hit’ model did not yield more pronounced aberrant drinking behavior. Similarly, when challenged acutely with amphetamine, Simpson et al. [50], Ashby et al. [51] and Hickey et al. [24] failed to observe a ‘double hit’ effect on locomotor activity. Taken together, these results provide little support for the ‘double-hit’ as a more robust model of schizophrenia-like symptoms.

NMDA receptor-antagonist pretreatment in this study produced a small but non-significant increase in SIP (Fig. 2A). Blunted SIP may have been due to the possible neuroprotective effects of group housing in the present study. Animals in the Hawken et al. [18] study were individually housed upon arrival (P46–52) and throughout the study; in the present study animals arrived at P21 and remained group housed. Alternately, failure to demonstrate a significant effect of MK-801 in this study may have also been due to other inconsistencies either in or during injection procedures between the two studies, for example, age of animals or colonization of vivarium during the studies. Finally, failure to significantly replicate the MK-801 effect in this study may simply be due to a lack of power.

The development of SIP is highly variable across animals [41], [52] which may in part result from neurochemical differences between individuals [42]. Jones et al. [53] reported *decreased* SIP in female rats following a social isolation protocol. Social isolation has been reported to induce sex-specific behavioral responses, increasing activity in female rats compared to males [54]. Jones et al. [53] reported that female rats showed significant hyperactivity during the SIP paradigm. Increased locomotor activity in the operant chamber could account for the observed decrease in SIP acquisition. As primary polydipsia is significantly associated with being male [1], [55], employing male rats to model the illness may be preferable.

This study investigated the impact of two animal models of schizophrenia-like symptoms (isolation rearing and subchronic MK-801 pretreatment) on drinking behavior in a SIP paradigm. The findings that post-weaning social isolation rearing significantly increased SIP suggests that a putative schizophrenic-like neuropathology resulting from isolation rearing sets the stage for the development of disordered water drinking. The deficits, neuroanatomical correlates, and mechanisms that contribute to the development of SIP from both social isolation and schizophrenia, however, require further investigation.
